# A rare gastric metastasis secondary to residual cystic duct carcinoma: case report and literature review

**DOI:** 10.1093/jscr/rjac593

**Published:** 2022-12-30

**Authors:** Leimin Qian, Jianming Huang

**Affiliations:** Department of Gastrointestinal Surgery, Jiangyin People's Hospital Affiliated to Southeast University, Jiangyin, Jiangsu, China; Department of Gastrointestinal Surgery, Jiangyin People's Hospital Affiliated to Southeast University, Jiangyin, Jiangsu, China

**Keywords:** residual cystic duct carcinoma, gastric metastasis, gastric cancer

## Abstract

An unusual gastric metastasis from residual cystic duct carcinoma was reported, which was easily mistaken as primary gastric carcinoma before the surgery. A 50-year-old Chinese man presented with right upper abdominal discomfort. Based on the biopsy and computed tomography results, an advanced gastric antrum adenocarcinoma was primarily diagnosed. Intraoperatively, there were other findings: residual cystic duct with chronic hyperplasia, a suspected purulent cavity filled with grayish-brown cloudy liquid at the distal end of the cystic duct and the gallbladder socket. The patient underwent radical operation. Histopathological findings finally suggested that adenocarcinoma of the residual cystic duct infiltrated into the whole layer of the gastric wall. Postoperative adjuvant chemotherapy and immunotherapy were administered. The patient has achieved 20-month recurrence-free survival. The comprehensive treatment including radical surgery, adjuvant chemotherapy and immunotherapy may improve the prognosis of such patients.

## INTRODUCTION

Residual cystic duct is a common complication in the resection of benign lesions of the gallbladder, which was first reported by Florcken in 1912 [1]. It is commonly caused by incomplete cholecystectomy in initial operation with the residual length of the cystic duct greater than or equal to 1 cm. Some researchers have suggested that primary cancer of residual cystic duct should occurred after cholecystectomy at least 5 years or above [2]. Similar to gallbladder cancer, residual cystic duct carcinoma can directly invade adjacent organs, such as liver and extrahepatic bile ducts, but the case of invading the stomach is relatively rare. Here, a case was reported in which gastric metastasis from residual cystic duct carcinoma mimicked primary gastric cancer.

## CASE REPORT

**Figure 1:**
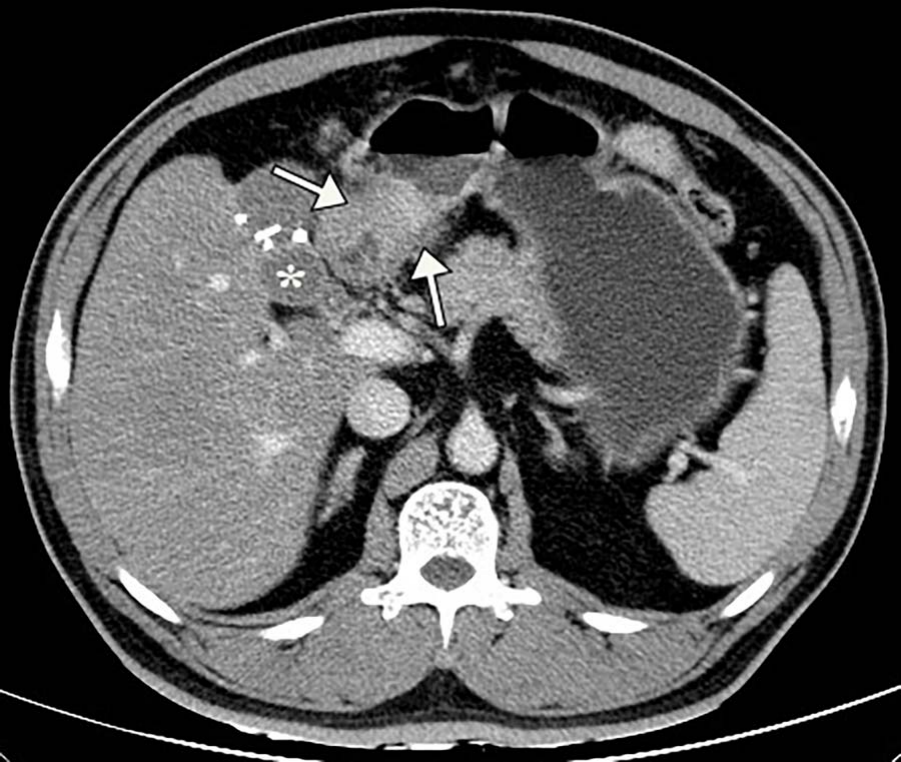
Preoperative contrast abdominal CT scan showed the thickening antral wall with uneven enhancement (white arrows) and a cystic foci (white asterisk) in the gallbladder bed with hyperdense clips from previous cholecystectomy.

**Figure 2:**
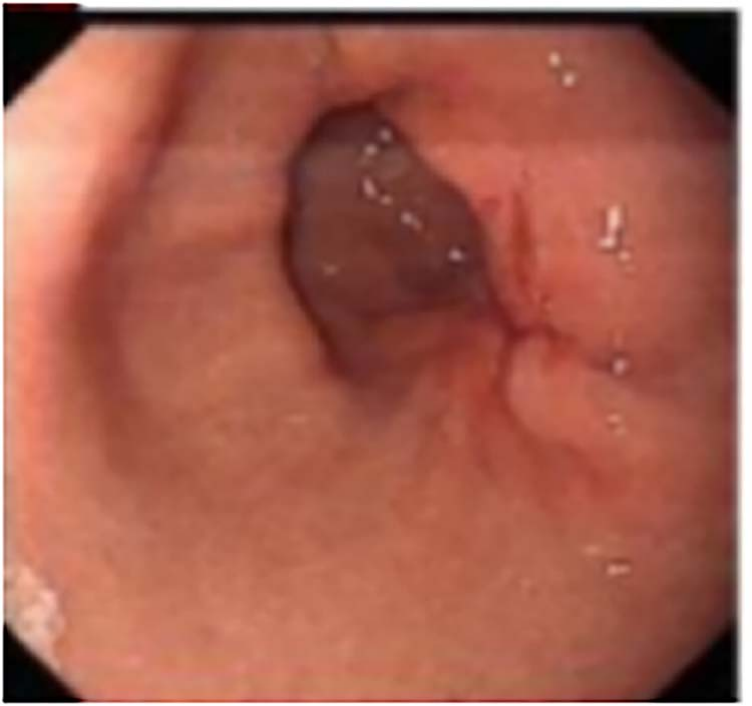
Endoscopy revealed the mucosa of gastric antrum was rough, with convergence of the surrounding folds and deformation on the large curved side of the posterior wall. At the oral side, there was a gentle slope uplift under the mucosa, about 0.6 × 0.8 cm in size.

A 50-year-old Chinese man presented with right upper abdominal discomfort for 18 days. A computed tomography (CT) enhanced scan showed the thickened wall of the antrum and a cystic foci in the gallbladder bed (Fig. 1). Endoscopy revealed submucosal tumor with ulcer formation situated at the gastric antrum (Fig. 2). Pathology was positive for adenocarcinoma. His past history included hypertension, type 2 diabetes, hyperuricemia and hyperlipidemia. He had a medical history of laparoscopic cholecystectomy, performed 20 years ago for acute gangrenous cholecystitis. Physical examination revealed mild right upper epigastric tenderness.

On examination, abnormal results were as follows: glutamyl transpeptidase 58 IU/L (normal; 7–47 IU/L), triglyceride 4.22 mmol/L (0.45–1.65 mmol/L), apolipoprotein B 1.52 g/L (0.60–1.10 g/L) and glucose 8.73 mmol/L (3.60–6.10 mmol/L). Carbohydrate antigen 19-9 (CA19-9) was elevated to 41.5 U/mL. Based on the biopsy and CT results, an advanced gastric antrum adenocarcinoma was primarily diagnosed. The patient was to undergo radical distal gastrectomy operation.

Intraoperatively， the length of the residual cystic duct was about 2.0 cm, and the wall of the residual cystic duct was chronic hyperplasia. A suspected purulent cavity filled with grayish-brown cloudy liquid was found at the distal end of the residual cystic duct and the gallbladder socket. The gastric lesion was located near the greater curvature of the anterior wall of gastric antrum, with a diameter of about 2.0 cm, corresponding to dense adhesion between the serosal surface and the above purulent cavity wall, and several lymph nodes around the stomach were enlarged. We performed radical distal gastrectomy (Billroth Ι type), residual cysticoectomy, abdominal abscess incision and drainage, and standard D2 lymphadenectomy.

**Figure 3:**
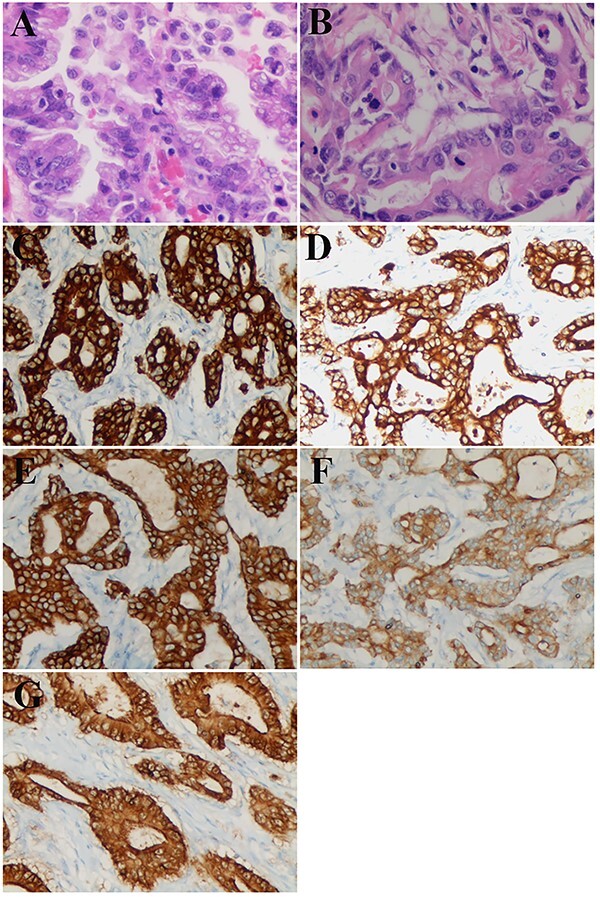
Pathological and immunostaining findings. (**A**) Remnant cystic duct adenocarcinoma, hematoxylin–eosin staining (×40). (**B-G**) Adenocarcinoma of the antrum at the greater curvature: (B) hematoxylin–eosin staining (×40). (C) CKAE1 staining (×20): positive. (D) CK7 staining (×20): positive. (E) CK8/18 staining (×20): positive. (F) CK19 staining (×20): weakly positive. (G) Villin staining (×20): positive.

Pathological examination showed that adenocarcinoma of the residual cystic duct infiltrated into the whole layer of the gastric wall. Carcinoma nodules around the outer serosal wall of the gastric wall and one lymph node metastasis in the minor curve were detected. Immunohistochemical stains showed that the antrum hyperplastic epithelial tissue was positive for CKAE1/AE3， CK7， CK8/18， CK19， CDX2， Villin and negative for CK20 (Fig. 3). Remnant cystic duct hyperplasia epithelial tissue was positive for CDX2, Villin and also negative for CK20.Based on these findings, the final diagnosis was residual cystic duct adenocarcinoma with gastric metastasis, and the tumor staging was pT3N1M0, Stage IIIB as per the eighth edition of AJCC/UICC. The patient declined additional surgery. His postoperative recovery course was uneventful and he was discharged on postoperative Day 16. One month after the surgery, chemotherapy with oxaliplatin, gemcitabine and immunotherapy with tislelizumab were administered. He remained asymptomatic post-operation but on followup, and was found to have rising tumor marker CA19-9 in recent 6 months. CT scan 20 months after the surgery revealed no obvious activity of recurrence or metastasis.

## DISCUSSION

Primary cancers of organs adjacent to the stomach, including gallbladder, extrahepatic bile duct, pancreas and transverse colon, can infiltrate the gastric wall through direct invasion [3–7]. In present case, the first impression was the high possibility of primary gastric cancer invading the perigastric organs. Other intraoperative findings included residual cystic duct and the surrounding abscess, since the previous operation was difficult due to gallbladder gangrene which resulted in anatomical disorganization and tissue edema. According to the results of immunohistochemical staining, the primary lesion was finally found to be located in the residual cystic duct, and the stomach was the metastatic organ. In 2016, Lo *et al*. [8] reported for the first time a case of pyloric obstruction caused by residual cystic duct carcinoma invading the pylorus and duodenal bulb, and the present study was the second case.

The biological characteristics of residual cystic duct carcinoma are similar to those of gallbladder cancer. The incidence of gallbladder cancer is relatively low, accounting for only 0.6% in all cholecystectomy specimens, and cystic duct cancer is about 0.44% [9, 10]. The occurrence of residual cystic duct carcinoma is even more rare. Most of the foreign literature reports are mainly necropsy or case reports (Table 1) [2, 8, 11–18]. The currently recognized diagnosis of residual cystic duct carcinoma must meet the criteria proposed by Ozden: the tumor center is located in the cystic duct [19]. The stimulation of long-term chronic inflammation may result in the course of carcinogenesis including intestinal metaplasia of cystic duct mucosa and markable dysplasia [20]. Some cases may be roughly judged from the external invasion course of cancer cells. Primary cancers of perigastric organs invade the gastric wall in the following order: mucosa of perigastric organ, submucosa layer, muscularis propria layer, serosa, gastric serosa, muscularis propria, submucosa and mucosa. So gastric mucosal lesions may be smaller than serous lesions, or normal [4, 5]. Postoperative immunohistochemical staining of specimens in detail is helpful to identify the primary lesion. Based on the immunohistochemical results, especially the positive markers CK19 and Ck7, we diagnosed metastatic gastric cancer caused by residual cystic duct carcinoma rather than primary gastric adenocarcinoma [4, 21]. Like for gallbladder cancer, the prognosis of residual cystic duct carcinoma is poor. However, the patient achieved relapse-free survival after chemotherapy and immunotherapy. The current tumor condition was evaluated as stable disease (SD) according to the clinical manifestations and imaging results.

**Table 1:** Summary of case reports of remnant cystic duct cancer

Serial number	Author/ year/ country	Age (y.o.)	Gender	Presentation	Etiology of previous cholecystectomy	T (yrs)	Histological type	Operation procedure	TNM staging	Visceral infiltration	Outcome (month)
1	Kuwayti *et al*. 1957 (USA) [11]	73	Female	ND	Acute cholecystitis	3	ND	Autopsy	ND	ND	ND
2	Phillips *et al*. 1969 (USA) [12]	57	Male	Abdominal pain, jaundice	Cholelithiasis	6	AC	Biopsy and T-tube drainage	ND	Liver metastases	ND
3	Dixon *et al*. 1971 (USA) [13]	77	Female	Abdominal pain, loss of weight	Chronic cholecystitis with gallstone	5	muc	Biopsy and T-tube drainage	ND	Involvement of RHA,PV	ND
4	Gabata *et al*. 2003 (Japan) [14]	70	Male	Abdominal pain, fever, jaundice	Cholecystolithiasis	0.5	pap	PD	T3NxMx	Involvement of the pancreatic duct	ND
5	Noji *et al*. 2003 (Japan) [2]	62	Male	Abdominal discomfort	Cholelithiasis	15	Tub2-por	ERH, BD, Co	T3N2M0	Involvement of the transverse colon	6; NED
6	Eum *et al*. 2008 (Korea) [15]	45	Male	None	Cholelithiasis	20	por	BD	T3N0M0	Involvement of the duodenum	6; NED
7	Do *et al*. 2014 (Korea) [16]	74	Male	Abdominal pain	Acute cholecystitis with gallstone	10	AC	Complete excision of the remnant cystic duct, wedge segment IVb and V and lymphadenectomy	T2N0M0	None	12; NED
8	Lo *et al*. 2006 (China) [8]	54	Male	Vomiting	Cholelithiasis	18	tub2	Complete excision of the remnant cystic duct，DG	T3N0M0	Involvement of duodenal bulb and pylorus	8; Liver metastases
9	Prasoon *et al*. 2019 (Japan) [17]	81	Female	Abdominal pain	Chronic cholecystitis with gallstone	2	tub1	BD	T1bN0M0	None	48; Death due to colon cancer
10	Yasuda *et al*. 2021 (Japan) [18]	81	Male	Loss of appetite	Acute cholecystitis	5	pap > tub2	BD	T1bN0M0	None	8; NED
11	Present case	50	Male	Abdominal discomfort	Acute gangrenous cholecystitis	20	AC	Complete excision of the remnant cystic duct，DG	T3N1M0	Involvement of gastric antrum	20;NED

ND: not documented, AC: adenocarcinoma, RHA: right hepatic artery, PV: portal vein, pap:papillary adenocarcinoma, PD: pancreatoduodenectomy, ERH: extended right hepatecyomy, BD: bile duct resection, Co: partial resection of the transverse colon, DG: distal gastrectomy, NED: (alive with) no evidence of disease.

In conclusion, we reported a case of gastric metastasis secondary to residual cystic duct carcinoma which was suspected of gastric cancer preoperatively. After radical distal gastrectomy together with residual cysticoectomy and postoperative adjuvant chemotherapy and immunotherapy, the patient has achieved recurrence-free survival. Our case report can provide a certain reference value for the comprehensive treatment of residual cystic duct carcinoma with gastric metastasis.

## CONFLICT OF INTEREST STATEMENT

We declare that we have no conflict of interest.

## FUNDING

None.

## DATA AVAILABILITY

The data of this case report are available from the corresponding author upon reasonable request.
